# A Digital Acceptance and Commitment Therapy and Education Intervention for Caregivers of Very Preterm Infants in the Neonatal Intensive Care Unit: Randomized Controlled Trial

**DOI:** 10.2196/92021

**Published:** 2026-07-23

**Authors:** Kristin Harrison Ginsberg, Jane Alsweiler, Jenny Rogers, Alana Cavadino, Meihana Douglas, Anna Serlachius

**Affiliations:** 1 Department of Paediatrics: Child and Youth Health, Faculty of Medical and Health Sciences, University of Auckland, 85 Park Rd, Grafton, Auckland, 1023, New Zealand, 64 (09) 923 5025; 2 Liggins Institute, University of Auckland, Grafton, Auckland, New Zealand; 3 Department of Epidemiology and Biostatistics, Faculty of Medical and Health Sciences, University of Auckland, Grafton, Auckland, New Zealand; 4 School of Clinical Sciences, Auckland University of Technology, Auckland, New Zealand; 5 Department of Psychological Medicine, Faculty of Medical and Health Sciences, University of Auckland, Grafton, Auckland, New Zealand

**Keywords:** neonatal intensive care unit, preterm infants, caregivers, digital mental health, acceptance and commitment therapy, stress, randomized controlled trial, digital health intervention

## Abstract

**Background:**

Parents of very preterm infants admitted to the neonatal intensive care unit (NICU) experience high levels of psychological distress, yet access to timely, evidence-based mental health support is limited by staffing and resource constraints. Digital mental health interventions offer a scalable approach to addressing this gap; however, their effectiveness has not been well established in NICU caregiver populations, particularly during periods of acute stress.

**Objective:**

This study aims to evaluate the effectiveness of a self-guided digital acceptance and commitment therapy (ACT)–based intervention combined with NICU-specific education (NICU parent acceptance and commitment therapy [NPACT]). The study explored the intervention’s effects on stress among parents and primary caregivers of very preterm infants, compared to a digital education-only intervention, and active control.

**Methods:**

We conducted a 3-arm, single-center, randomized controlled cluster trial in a tertiary NICU. Parents and primary caregivers of very preterm infants (<32 wk’ gestational age,<1 wk old) were randomized by family cluster to (1) NPACT (ACT+ education), (2) a digital education-only intervention, or (3) active control. Digital interventions were delivered via a web-based platform over 2 weeks. The primary outcome was NICU-related stress on the Parent Stressor Scale: Neonatal Intensive Care Unit (PSS:NICU) at 2 weeks postrandomization. Secondary outcomes included caregiver anxiety, depression, perceived stress, and selected neonatal outcomes. Engagement and perceived helpfulness were assessed for digital interventions.

**Results:**

A total of 102 caregivers from 68 family clusters (79 infants; mean gestational age 28.1, SD 2.2 wk) were enrolled. There were no statistically significant between-group differences in the mean PSS:NICU scores at 2 weeks (NPACT 3.0, SD 0.9; education-only 2.5, SD 1; active control 2.6, SD 0.9; adjusted mean difference for NPACT vs active control 0.04, 95% CI −0.39 to 0.47). No between-group differences were observed for secondary psychological outcomes at any time point. However, caregivers in both digital intervention groups had higher odds of full breastfeeding at discharge compared with active control. Engagement with the digital interventions was high, with 97% (28/29) of NPACT participants and 76% (19/25) of education-only participants completing at least 5 of 7 modules, and both interventions were rated as very helpful.

**Conclusions:**

In this trial, an unguided digital mental health intervention delivered during NICU admission did not reduce NICU-specific parental stress or other psychological outcomes relative to active control. However, the intervention was highly used by caregivers. These findings suggest that while a brief digital mental health intervention can be successfully implemented in a high-stress clinical setting with caregivers, its capacity to reduce acute psychological distress may be limited. Secondary findings indicate potential benefits of the digital intervention on breastfeeding, generating hypotheses for future research. Digital mental health interventions in neonatal settings may be most effective when integrated within hybrid models of care and/or delivered beyond the acute admission phase.

## Introduction

Parents of infants admitted to the neonatal intensive care unit (NICU) experience high levels of psychological distress, including elevated symptoms of anxiety, depression, and acute stress, compared with the general postpartum population [1-3]. Distress is particularly pronounced among caregivers of very preterm infants (<32 wk’ gestational age), whose infants face increased risks of mortality and severe neonatal morbidity, often requiring prolonged and medically complex NICU admissions [4]. Persistent parental distress during NICU hospitalization has been associated with adverse outcomes for both caregivers and infants, including impaired parent–infant bonding and attachment and challenges with parenting behaviors following discharge [3,5-7].

Although effective psychosocial and psychological interventions exist to reduce caregiver distress in the NICU, implementation remains inconsistent [3,5-7]. Many NICU-based interventions rely on intensive, face-to-face delivery models that require substantial staff time, training, and funding, limiting scalability and equitable access [7]. Recent surveys conducted in New Zealand, Australia, and the United States have demonstrated that psychosocial support services for caregivers in the NICU are often under-resourced, with limited availability of trained mental health professionals and inconsistent screening and follow-up practices [8,9]. These structural constraints highlight the need for alternative models of mental health support that can be delivered efficiently within resource-limited neonatal care settings.

Digital mental health interventions offer a potentially scalable solution to these challenges by enabling flexible, low-cost delivery of psychological support [10]. In pregnant and postpartum populations, digital mental health interventions have been shown to reduce symptoms of anxiety and depression and are generally acceptable to users [11,12]. Despite the high prevalence of distress among caregivers and their limited access to psychosocial services in the NICU, digital mental health interventions have rarely been evaluated among parent populations in the NICU, particularly using randomized controlled designs.

Acceptance and commitment therapy (ACT) is a transdiagnostic, evidence-based psychological intervention that emphasizes mindfulness, acceptance of difficult internal experiences, and engagement in values-based actions [13,14]. ACT has demonstrated effectiveness in reducing distress among parents of children with chronic health conditions [15] and has been successfully adapted for digital delivery and in brief formats [16]. ACT has been proposed as a particularly suitable approach for populations facing ongoing or uncontrollable stressors, as it prioritizes psychological flexibility rather than symptom elimination [13,14].

Prior qualitative work conducted to inform the present trial found that caregivers and NICU clinicians expressed a strong desire for accessible, context-specific digital resources. Participants emphasized the importance of interventions that are brief, relevant to the NICU experience, and flexible enough to fit within the demands of acute neonatal care [17]. These findings informed the design of this trial’s intervention to support acceptability and engagement, and they influenced the selection of ACT as an appropriate psychological modality for use in the intervention.

The digital intervention used in this trial, NICU parent acceptance and commitment therapy (NPACT) plus education, combined evidence-based, brief ACT-based psychological strategies with NICU-specific educational content. The intervention was intentionally designed to be self-paced and unguided, minimizing reliance on clinical staff and maximizing potential scalability within resource-constrained neonatal settings.

The primary aim of this randomized controlled cluster trial was to evaluate whether the NPACT digital intervention reduced NICU-related stress among caregivers of very preterm infants more than a digital education-only intervention or active control. Caregivers of very preterm infants were selected for this trial because they are an underrepresented group in intervention research, despite this population experiencing high levels of NICU-related stress and prolonged exposure to the intensive care environment [7].

Secondary aims were to assess the effects of the NPACT intervention on other caregiver psychological outcomes, including perceived stress, anxiety, depression, and psychological flexibility, as well as on infant outcomes, including breastfeeding at discharge, length of stay, and selected neonatal morbidity indicators. We examined changes in outcomes over time: at 2 weeks postrandomization, at discharge, and 3 months after discharge. We hypothesized that caregivers receiving the NPACT intervention would report lower NICU-specific stress compared to the education-only group or those receiving active control.

## Methods

### Study Design

This study was a single-center, 3-arm, randomized controlled cluster trial conducted in a tertiary-level NICU. Family clusters were randomized to one of three conditions: (1) a digital acceptance and commitment therapy–based intervention combined with NICU-specific education (NPACT), (2) a digital education-only intervention, or (3) active control. Cluster randomization by family was used to minimize contamination between caregivers of the same infant. The trial was prospectively registered with the Australian New Zealand Clinical Trials Registry (ACTRN12623000641695p) and the protocol published [18]. It is reported in accordance with CONSORT (Consolidated Standards of Reporting Trials) guidelines, including extensions relevant to cluster randomized trials [19] and digital health interventions [20].

### Participants and Recruitment

Parents and primary caregivers of very preterm infants admitted to the NICU were eligible for participation. Inclusion criteria were: (1) infant gestational age <32 weeks, (2) infant age <1 week at the time of recruitment, and (3) caregiver ability to provide informed consent. Caregivers were excluded if they were unable to complete study procedures due to language barriers or cognitive impairment. Eligible caregivers were identified by clinical staff and approached by a member of the research team during the infant’s NICU admission. Written informed consent was obtained from all participants prior to enrollment.

### Randomization and Allocation

Randomization occurred at the family cluster level following consent. Clusters were allocated in a 1:1:1 ratio to the 3 study arms using a computer-generated randomization schedule. Allocation concealment was maintained until assignment. Due to the nature of the interventions, participants and study personnel were not blinded to group allocation; however, outcome measures were self-reported using standardized online questionnaires, minimizing investigator influence.

### Interventions

#### Digital ACT-Based Intervention Plus Education (NPACT)

**Figure 1.**
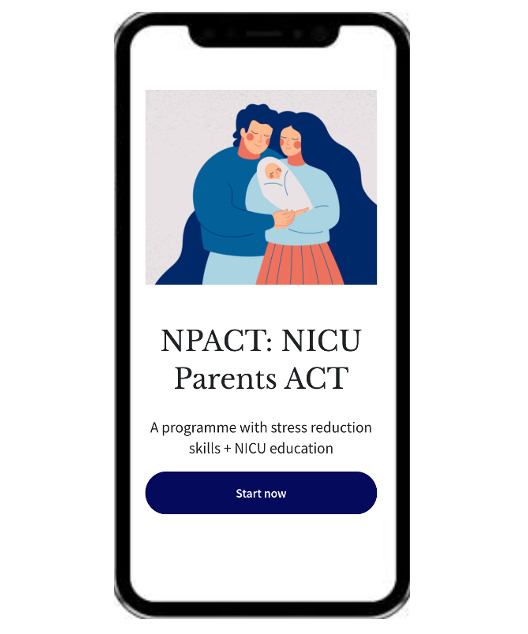
NICU Parent Acceptance and Commitment Therapy. intervention home screen. NICU: neonatal intensive care unit; NPACT: NICU Parent Acceptance and Commitment Therapy.

The NPACT intervention consisted of a self-guided, web-based program that combined brief Acceptance and Commitment Therapy (ACT)–based psychological activities with NICU-specific educational content (Figure 1). The intervention was delivered via an online learning platform (Thinkific.com) and structured into 7 modules designed to be completed over approximately 2 weeks. ACT components targeted mindfulness, acceptance of difficult internal experiences, cognitive defusion, values clarification, and values-based action, with content tailored to the NICU context. Seven educational modules provided information relevant to the care and development of a very preterm infant in the NICU.

The intervention was intentionally designed to be unguided and self-paced to reduce reliance on clinical staff and enhance scalability within this resource-limited NICU setting. Participants were able to access the intervention at any time using personal digital devices. (Find more details about the intervention in the published protocol [18].)

#### Digital Education-Only Intervention

Participants randomized to the digital education-only arm received access to the NICU-specific educational modules without the ACT-based psychological components. Content was delivered via the same online platform and over the same time frame as the NPACT intervention, allowing isolation of the added value of the ACT-based components.

### Active Control

Participants assigned to the active control arm received standard NICU care, which included access to existing psychosocial resources available at the study site. In addition, like the intervention groups, participants in the active control group were asked to complete a 7-module digital course, delivered via the same online platform as the intervention groups, that used content from the study site’s existing public web pages.

### Outcomes

The primary outcome was scores on the Parental Stressor Scale: neonatal intensive care unit (PSS:NICU; 27 items, with higher scores indicating higher stress [21]) at 2 weeks postrandomization. This timepoint was selected to measure outcomes at the conclusion of the 2-week intervention and to minimize confounders such as transfers to lower-level units of care.

Secondary caregiver outcomes were the three PSS:NICU Subscales [21]; the Perceived Stress Scale (PSS), a 10-item measure [22]; the Spielberger State-Trait Anxiety Index (STAI-6), the short version of the 20-item State-Trait Anxiety Index-State (STAI-S) [23]; the Center for Epidemiologic Studies Depression Scale (CESD-10), the short form of the 20-item scale [24]; and the Acceptance and Action Questionnaire-9 (AAQ-9), which measures psychological flexibility, the main psychological process involved in ACT-based interventions [25]. Outcomes were collected at 2 weeks after randomization, discharge to home, and 3 months postdischarge.

Neonatal morbidity data were collected from the medical record at discharge to home. Data collected included: the occurrence of intraventricular hemorrhage, necrotizing enterocolitis, late-onset sepsis treated with antibiotics for more than 5 days, chronic lung disease, retinopathy of prematurity requiring treatment, transfer to a Level 2 unit at another hospital, length of stay, full breastfeeding at discharge (defined as fully breastfed with no supplementation for 24 hours or ≥150 ml.kg.-1d-1 expressed breastmilk by bottle), and if parents received extra support from the social work or mental health team.

Intervention adherence was measured using activity log data provided by the intervention website platform Thinkific.com. Additionally, participants were asked to complete brief Likert-scale surveys on the content’s helpfulness (1=“not at all helpful” to 5=“extremely helpful”) at the end of each module and at the end of the intervention.

### Sample Size Calculation

We conducted a power calculation to detect a reduction in the individual total PSS:NICU stress score from 2 to 1.8, with an SD of 0.23, at 80% power and a 2-sided alpha of .05. This large effect size was derived from a between-group improvement in parental stress scores on the PSS:NICU reported in a previous RCT on parental stress in the NICU [26].

We predicted a mean cluster size of 1.7 participants per family unit (based on a study with similar eligibility criteria at the same study site [13]) and used an intracluster correlation (ICC) of ρ=0.5, which was the average ICC between parents of hospitalized children reported in a previous study [27]. We added 12% to our sample size to account for potential participant dropout rates [7] and early neonatal death, leading to a total sample size of 102 participants from 60 family units.

### Statistical Analysis

The primary analysis population was based on the modified intention-to-treat (ITT) principle and included all participants in the groups to which they were assigned at randomization. Participants were excluded from the ITT population only if they did not satisfy the entry criteria (ie, they were randomized in error) or if their infants died before the primary outcome measure timepoint. Exclusion was necessary because the primary outcome (PSS:NICU) could not be assessed following infant death.

Adult participant outcomes were analyzed using linear mixed-effects regression models, adjusted for baseline scores, stratification factor (multiple pregnancies), and a random effect for family (cluster). Between-group differences and change over time differences, both for the full cohort and within groups, were estimated with a 95% CI. A value of *P*<.05 was considered statistically significant.

A per-protocol analysis was also conducted for all participants who were not transferred to another hospital within 2 weeks after birth, and for those participants in the intervention groups (NPACT and education-only) who completed at least 5 out of 7 intervention modules.

Neonatal morbidity outcomes at discharge could not be analyzed using linear mixed-effects models due to high within-twin correlation and small sample sizes, which prevented model convergence. Instead, we accounted for the non-independence of twin pairs using multiple outputation [28,29], an approach used in similar studies with twins and preterm infants [30]. An overall multiple outputation regression estimate and its variance are reported.

We analyzed the intervention adherence and helpfulness quantitative data using descriptive statistics. Open-ended comments describing participant experience were analyzed using directed content analysis, a qualitative method guided by predetermined research questions and existing theory [31] and recommended for digital health intervention studies [32].

Secondary outcomes were prespecified, intended to be hypothesis-generating, and interpreted cautiously; no formal adjustment for multiple comparisons was applied.

### Ethical Considerations

Ethical approval was provided by the New Zealand Health and Disability Ethics Committee (#2023 EXP 17879). All participants provided written informed consent prior to participation. Participants received NZD $20 (NZD $1 = US $0.62 as of Dec 15, 2023) for completing the baseline measures and NZ $90 for completing subsequent follow-up measures at 3 additional timepoints. The maximum koha (token of thanks for their time) was NZ $110 over the study time period.

## Results

### Participant Flow and Retention

**Figure 2.**
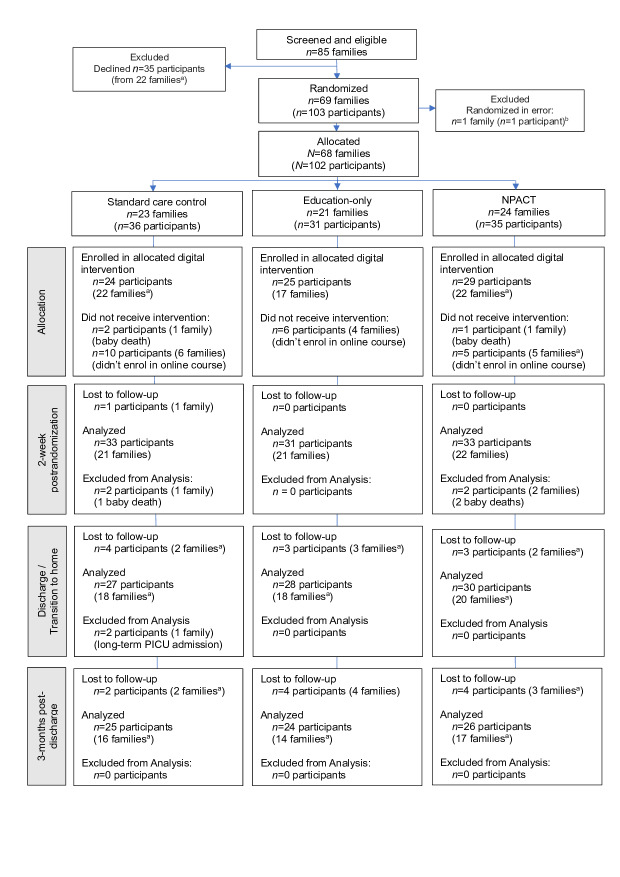
CONSORT (Consolidated Standards of Reporting Trials) flow diagram. NPACT: NICU parent acceptance and commitment therapy+ education intervention. ^a^Some families had one member enroll and/or complete follow-up while the other member did not. ^b^randomized in error due to the death of a twin before randomization.

Between December 2023 and October 2024, 102 participants from 68 family clusters were recruited and enrolled in the trial (Figure 2). Follow-up data collection was completed in March 2025.

Retention was high at the primary outcome timepoint, with 97 participants (95%) completing assessments at 2 weeks postrandomization. Overall, 85 participants (83%) completed outcome measures at discharge to home, and 75 participants (74%) completed assessments at 3 months postdischarge.

Attrition differed by caregiver role. Fathers had a higher drop-out rate than mothers (30% vs 17%, *P*=.03).

### Participant and Infant Characteristics

Baseline characteristics of participants and their infants are presented in Table 1. Most participants were mothers (66/102, 65%), followed by fathers (33/102, 32%) and other caregivers (3/102, 3%). The cohort was ethnically diverse, with participants identifying as European (29/102, 29%), Indigenous Māori (13/102, 13%), Pacific Peoples (22/102, 22%), Indian (20/102, 20%), and other ethnic groups.

**Table 1.** Characteristics of participants and related babies.

Characteristics	Active control	Education-only	NPACT^a^	Total
Parent and caregiver				
Number of participants	36	31	35	102
Sex, n (%)				
Female	21 (58)	22 (71)	25 (71)	68 (67)
Caregiver role, n (%)				
Mother	21 (58)	21 (68)	24 (69)	66 (65)
Father	14 (39)	9 (29)	10 (29)	33 (32)
Whānau extended family member	1 (3)	1 (3)	1 (3)	3 (3)
Ethnicity (prioritized), n (%)				
Māori	4 (11)	4 (13)	5 (14)	13 (13)
Pacific	8 (22)	10 (32)	4 (11)	22 (22)
Indian	7 (19)	3 (10)	10 (29)	20 (20)
European	13 (36)	7 (23)	9 (26)	29 (28)
Other Asian	3 (8)	2 (7)	4 (11)	9 (9)
MEELA^b^ and others	1 (3)	5 (16)	3 (9)	9 (9)
Age, years, mean (SD)	35.2 (8.8)	33.5 (8.6)	34.2 (6.1)	34.3 (7.9)
Employed, n (%)	31 (86)	22 (71)	26 (74)	78 (78)
Level of education, n (%)				
Primary school	0 (0)	2 (7)	0 (0)	2 (2)
Secondary school	13 (36)	11 (36)	6 (17)	30 (29)
University	11 (31)	7 (23)	15 (43)	33 (32)
Postgraduate	7 (19)	6 (19)	12 (34)	25 (25)
Unknown	5 (14)	5 (16)	2 (6)	12 (12)
Marital Status, n (%)				
Single (never married)	0 (0)	2 (7)	1 (3)	3 (3)
Living with partner	5 (14)	10 (32)	6 (17)	21 (21)
Married	24 (67)	18 (58)	26 (74)	67 (67)
Divorced or prefer not to answer^c^	7 (19)	1 (3)	2 (6)	10 (10)
Mental health history				
Prior history of anxiety or depression	11 (31)	5 (16)	9 (26)	25 (25)
Currently taking medications for anxiety or depression	4 (11)	0 (0)	4 (11)	10 (10)
Above screening threshold^d^, n (%)				
Anxiety	19 (54)^d^	15 (48)	22 (65)^e^	56 (56)^f^
Depression	18 (50)^d^	13 (42)	18 (51)^e^	49 (49)^f^
Both anxiety and depression	14 (38)^d^	10 (32)	15 (43)^e^	39 (39)^f^
Baby characteristics				
Number of babies	27	24	28	79
Maternal antenatal events				
Maternal diabetes	6 (23)	4 (17)	2 (7.1)	12 (16)
Maternal antenatal corticosteroids	22 (92)	22 (96)	28 (100)	72 (96)
Mode of birth, n (%)				
Nonassisted vaginal	11 (41)	7 (29)	9 (32)	27 (34)
Instrumental vaginal	0 (0)	2 (8)	0 (0)	2 (3)
Elective cesarian	7 (26)	2 (8)	6 (21)	15 (19)
Emergency cesarian	9 (33)	13 (54)	13 (46)	35 (44)
Gestational age at birth, mean (SD), weeks	27.8 (2.3)	28.6 (1.8)	28 (2.3)	28.1 (2.2)
<28 weeks, n (%)	14 (52)	14 (58)	13 (46)	41 (52)
28 to <32 weeks, n (%)	13 (48)	10 (42)	15 (54)	38 (48)
Twin, n (%)	8 (30)	6 (25)	8 (29)	22 (28)
Baby’s sex (female), n (%)	11 (41)	10 (42)	21 (75)	42 (53)
Baby’s ethnicity (prioritized), n (%)				
Māori	4 (15)	7 (29)	3 (11)	14 (18)
Pacific	7 (26)	6 (25)	3 (11)	16 (20)
Indian	4 (15)	1 (4)	7 (25)	12 (15)
European	8 (30)	3 (13)	7 (25)	18 (22)
Chinese and other Asian	2 (7)	3 (13)	3 (11)	8 (10)
MEELA and others	2 (7)	4 (17)	5 (18)	11 (14)
Birth weight, grams, mean (SD)	1149.9 (359.2)	1226.5 (370.8)	1076 (412.6)	1146.9 (382.4)
CRIB^d^ II score, mean (SD)	7.7 (4)	7.2 (3)	8.1 (3.2)	7.7 (3.4)

a NPACT: NICU parent acceptance and commitment therapy + education intervention.

b MEELA: Middle Eastern, Latin American, African.

c To keep anonymity, categories merged due to low numbers in one group.

d CRIB: clinical risk index for babies.

Of the 79 infants, 53% (42/79) were female and 72% (57/79) were singletons. More than half of the infants (41/79, 52%) were born extremely preterm (<28 wk’ gestational age). Mean gestational age at birth was 28.1 (SD 2.2) weeks.

### Primary Outcome: Parental Stress in the NICU

At 2 weeks postrandomization, there was no statistically significant difference in total PSS:NICU scores between the NPACT group and active control (adjusted mean difference 0.04; 95% CI −0.39 to 0.47; *P*=.85). No significant differences were observed between the education-only group and active control, or between the 2 intervention groups (Table 2).

Per-protocol analyses yielded similar findings, with no between-group differences detected for the primary outcome (Table S1 in Multimedia Appendix 1).

**Table 2.** Summary statistics and linear mixed-model analysis of primary and secondary outcomes (between groups). Outcomes analyzed using linear mixed regression models with adjustment for scores at baseline, stratification factor (twin pregnancy), and a random effect for within-cluster (family) correlation.

Outcomes	Scores, Mean (SD), points						Difference between groups							
Outcome, timepoint	Active control	n	NPACT^a^	n	Education-only	n	NPACT vs active control M adjusted difference (95% CI)	*P* value	Adjusted Cohen *d*	Education-only vs active control M adjusted difference (95% CI)	*P* value	Adjusted Cohen *d*	NPACT versus Education-only M adjusted difference (95% CI)	*P* value
Primary outcome														
NICU Stress (PSS:NICU)^b^														
Baseline	2.3 (0.8)	36	2.8 (0.8)	34	2.3 (0.9)	31	—^c^	—	—	—	—	—	—	—
2 weeks	2.6 (0.9)	33	3.0 (0.9)	33	2.5 (1.0)	31	0.04 (−0.39 to 0.47)	*.*85	0.07	−0.04 (−0.47 to 0.40)	*.*87	−0.05	0.08 (−0.36 to 0.51)	.73
Secondary outcomes														
PSS:NICU^d^ Subscale 1														
Baseline	1.8 (0.7)	36	2.3 (0.7)	34	1.8 (0.9)	31	—	—	—	—	—	—	—	—
2 weeks	2.2 (1.4)	33	2.5 (1.7)	33	2.2 (1.7)	31	0.06 (−0.32 to 0.44)	*.*76	0.08	0.09 (−0.34 to 0.52)	*.*67	0.11	0.03 (−0.54 to 0.60)	.92
PSS:NICU^e^ Subscale 2														
Baseline	2.4 (1.0)	36	2.9 (1.0)	34	2.3 (1.3)	31	—	—	—	—	—	—	—	—
2 weeks	2.7 (1.0)	33	3.1 (1.1)	33	2.4 (1.0)	31	0.13 (−0.34 to 0.59)	*.*60	0.18	−0.14 (−0.61 to 0.33)	*.*56	−0.20	−0.05 (−0.72 to 0.61)	.87
^c^PSS:NICU^f^ Subscale 3														
Baseline	2.7 (1.0)	36	3.1 (1.0)	34	2.8 (1.1)	31	—	—	—	—	—	—	—	—
2 weeks	2.9 (1.9)	33	3.1 (1.7)	33	2.8 (1.4)	31	−0.07 (−0.62 to 0.48)	*.*80	−0.11	0.07 (−0.48 to 0.63)	.80	0.12	−0.2 (−0.77 to 0.34)	.49
Perceived Stress (PSS)														
Baseline	18.3 (6.5)	35	20.4 (6.6)	34	18.3 (6.3)	31	—	—	—	—	—	—	—	—
2 weeks	19.8 (6.0)	33	21.3 (6.0)	33	18.6 (5.8)	31	0.68 (−2.40 to 3.77)	*.*66	0.14	−0.69 (−3.84 to 2.45)	.66	−0.14	1.38 (−1.75 to 4.5)	.39
Discharge	17.0 (7.8)	27	17.4 (6.7)	30	17.9 (6.6)	28	0.19 (−3.07 to 3.45)	*.*91	0.04	1.82 (−1.51 to 5.15)	.28	0.37	−1.63 (−4.88 to 1.62)	.32
3-months	16.5 (7.1)	25	15.7 (7.4)	26	15.8 (6.3)	24	−1.03 (−4.44 to 2.37)	*.*55	−0.21	0.02 (−3.46 to 3.50)	.99	0	−1.05 (−4.50 to 2.39)	.55
Anxiety (STAI-6)														
Baseline	44.8 (16.9)	35	49.7 (13.0)	34	43.5 (14.1)	31	—	—	—	—	—	—	—	—
2 weeks	45.5 (14.6)	33	46.8 (13.8)	33	41.0 (16.1)	31	−1.03 (−8.05 to 5.99)	*.*77	−0.09	−3.24 (−10.38 to 3.90)	*.*37	−0.29	2.21 (−4.94 to 9.36)	.54
Discharge	41.3 (15.2)	27	37.9 (13.5)	30	40.5 (14.3)	28	−4.45 (−11.86 to 2.96)	*.*24	−0.40	1.36 (−6.22 to 8.93)	*.*73	0.12	−5.81 (−13.24 to 1.62)	.13
3-months	39.7 (16.3)	25	38.2 (14.4)	26	38.6 (14.2)	24	−3.15 (−10.90 to 4.60)	*.*43	−0.28	0.44 (−7.49 to 8.36)	*.*91	0.04	−3.58 (−11.47 to 4.31)	.37
Depression (CESD-10)														
Baseline	9.6 (5.8)	35	11.9 (6.0)	34	10.1 (6.1)	31	—	—	—	—	—	—	—	—
2 weeks	10.0 (6.0)	33	10.3 (6.0)	33	8.6 (5.9)	31	−0.69 (−3.12 to 1.74)	*.*58	−0.18	−1.34 (−3.80 to 1.13)	*.*28	−0.34	0.65 (−1.82 to 3.11)	.61
Discharge	9.5 (6.3)	27	8.8 (4.9)	30	9.1 (6.1)	28	−1.43 (−4.01 to 1.15)	*.*27	−0.37	−0.45 (−3.07 to 2.18)	*.*73	−0.11	−0.99 (−3.56 to 1.58)	.45
3-months	8.9 (5.8)	25	7.7 (6.1)	26	7.6 (5.5)	24	−2.26 (−4.97 to 0.44)	*.*10	−0.58	−1.29 (−4.04 to 1.46)	*.*39	−0.33	−0.97 (−3.70 to 1.76)	.48
Psychological Flexibility (AAQ-9)														
Baseline	32.0 (7.3)	35	34.1 (8.4)	34	32.3 (6.9)	31	—	—	—	—	—	—	—	—
2 weeks	33.5 (5.6)	31	34.7 (6.8)	32	30.8 (7.0)	29	0.33 (−3.12 to 3.77)	*.*85	0.06	−1.89 (−5.45 to 1.66)	*.*30	−0.35	2.22 (−1.30 to 5.74)	.22
Discharge	34.8 (6.6)	27	32.2 (8.1)	28	31.3 (7.2)	23	−2.36 (−6.03 to 1.31)	*.*21	−0.45	−0.13 (−4 to 3.74)	*.*95	−0.02	−2.23 (−6.04 to 1.58)	.25
3-months	34.7 (8.9)	23	33.2 (9.1)	25	32.5 (10.4)	22	−1.78 (−5.63 to 2.06)	*.*36	−0.34	−0.25 (−4.24 to 3.75)	*.*90	−0.05	−1.54 (−5.47 to 2.40)	.44

a NPACT: NICU parent acceptance and commitment therapy + education intervention.

b PSS:NICU: Parental Stressor Scale: neonatal intensive care unit.

c Not available.

d PSS:NICU Subscale 1 = sights and sounds of the NICU.

e bPSS:NICU Subscale 2 = baby behaviour and appearance.

f PSS:NICU Subscale 3 = parental role alteration.

### Secondary Caregiver Mental Health Outcomes

No statistically significant between-group differences were observed for any secondary caregiver outcomes at any assessed timepoint, including PSS:NICU subscales, perceived stress, anxiety, depression, or psychological flexibility (Table 2).

### Change Over Time in Caregiver Distress

In secondary within-group analyses, all 3 groups demonstrated significant increases in NICU-related stress from baseline to 2 weeks postrandomization (Table S2 in Multimedia Appendix 1). Over longer follow-up, reductions in perceived stress, anxiety, and depressive symptoms were observed within groups, although patterns varied by group, outcome, and timepoint. All within-group analyses are presented in Table S2 in Multimedia Appendix 1.

### Infant Clinical and Discharge Outcomes

Neonatal morbidity and discharge outcomes are summarized in Table 3. Neonatal outcomes were preidentified and examined as secondary, hypothesis-generating analyses.

Compared with active control, the odds of full breastfeeding at discharge were significantly higher in both intervention groups. The odds ratio for full breastfeeding at discharge was 3.75 (95% CI 1.08 to 13.07; *P*=.04) for the NPACT group and 4.37 (95% CI 1.21 to 15.81; *P*=.02) for the education-only group.

Infants in the NPACT group also had statistically lower odds of chronic lung disease compared with active control (OR 0.22; 95% CI 0.06 to 0.77; *P*=.02). No statistically significant between-group differences were observed for other neonatal morbidity or discharge outcomes (Table 3).

**Table 3.** Infant neonatal morbidity and discharge outcomes. No cases of retinopathy of prematurity requiring treatment were reported.

Outcomes	Active control (n=27 babies)	NPACT^a^ (n=28 babies)					Education-only (n=24 babies)			Total (n=79 babies)
	n (%)	n (%)	OR or MD (95% CI)	*P* vs active control	OR or MD (95% CI)	*P* vs education-only	n (%)	OR or MD (95% CI)	*P* vs active control	n (%)
Death^b^	1 (4)	2 (8)	—^c^	—	—	—	0	—	—	3 (4)
Intraventricular hemorrhage	10 (38)^d^	4 (16)^e^	0.23 (0.05 to 1.01)	.05	0.51 (0.10 to 2.45)	*.*40	5 (21)	0.45 (0.12 to 1.68)	*.*24	18 (24)
Necrotizing enterocolitis	0	1 (4)	—	—	0.29 (0.03 to 2.99)	*.*30	3 (13)	3.50 (0.33 to 36.67)	.30	4 (5)
Late-onset sepsis	4 (15)	1 (4)	0.21 (0.02 to 2.09)	*.*19	0.12 (0.01 to 1.09)	*.*06	6 (25)	1.80 (0.43 to 7.59)	.42	11 (15)
Chronic lung disease	17 (65)	7 (28)	0.22 (0.06 to 0.77)	.02	0.51 (0.15 to 1.77)	*.*29	11 (46)	0.42 (0.12 to 1.47)	.18	34 (45)
Parents extra social support	15 (58)	10 (40)	0.48 (0.14 to 1.59)	*.*23	0.63 (0.19 to 2.08)	*.*44	12 (50)	0.76 (0.22 to 2.59)	.66	36 (55)
Transferred	17 (77)	16 (72)	0.78 (0.20 to 3.08)	*.*73	0.75 (0.20 to 2.80)	*.*67	14 (67)	0.59 (0.15 to 2.3)	.44	47 (72)^f^
Discharge										
n (%)	26 (96)	25 (89)	—	—	—	—	24 (100)	—	—	75 (95)
Length of stay, days, mean (SD)	83.4 (29.1)	82.7 (31.7)	–6.6 (–26.4 to 13.2)	*.*51	0.3 (–19.7 to 20.3)	*.*98	81.6 (34.1)	–6.9 (–26.9 to 13.1)	.49	82.7 (31.2)
Full breastfeeding, n (%)	11 (42)	18 (72)	3.75 (1.08 to 13.07)	.04	0.86 (0.23 to 3.16)	*.*82	17 (71)	4.37 (1.21 to 15.81)	.02	46 (61)
Discharged with home oxygen, n (%)	10 (38)	4 (16)	0.32 (0.08 to 1.27)	.11	0.94 (0.20 to 4.39)	*.*94	4 (17)	0.34 (0.09 to 1.35)	.13	18 (24)
Weight (grams), mean (SD)	3550.7 (615.7)	3408.8 (623.2)	–165.1 (–519.5 to 327.2)	.65	–96.2 (–525.6 to 306.2)	*.*60	3485.5 (801.1)	–68.9 (–492.3 to 354.4)	.75	3482.52 (676)

a NPACT: NICU parent acceptance and commitment therapy + education intervention.

b All deaths occurred before the two-week intervention concluded.

c Not available.

d n=26 (1 infant death).

e n=25 (2 deaths and one infant removed due to twin death).

f Transfer numbers by family, n=68.

### Intervention Adherence and Acceptability

Engagement with both digital interventions was high. In the NPACT group, 28 of 29 participants (97%) completed at least 5 of the 7 intervention modules. In the education-only group, 19 of 25 participants (76%) completed at least 5 modules.

Participants rated both interventions as highly acceptable. Mean helpfulness ratings (on a scale from 1 “not at all helpful” to 5 “extremely helpful”) were 4.5 (SD 0.5) for the NPACT intervention and 4.7 (SD 0.4) for the education-only intervention.

Optional qualitative feedback on the interventions provided insights into caregiver experiences, perceived usefulness, and usability challenges. Qualitative results are presented in Table S3 in Multimedia Appendix 1.

## Discussion

### Principal Findings

This randomized controlled trial found that an unguided, brief, ACT-based digital intervention did not reduce NICU-related parental stress relative to active control during the early neonatal period. Null findings between groups were also found across secondary psychological outcomes, including depression, anxiety, and perceived stress. These results indicate that a brief, unguided digital mental health intervention delivered early in NICU admission may have limited capacity to influence psychological distress.

Caregivers of preterm infants experience rapidly fluctuating and often intensifying stress during early NICU admission, driven by medical uncertainty, infant instability, and evolving caregiving demands [33]. Under these conditions, psychological distress may represent an understandable and proportionate response to circumstances, and may be relatively resistant to short-term intervention, particularly when support is digital and unguided.

From an ACT theoretical perspective, the absence of change in psychological flexibility may help explain the lack of observed effects between groups on distress outcomes. ACT-based interventions aim to increase psychological flexibility, associated with reduced psychological distress, through mindfulness, cognitive defusion, and values-based action [13]. In this study, the absence of change may indicate that either the brief, unguided intervention was insufficient to shift psychological flexibility during an acutely stressful period, that the AAQ-9 was not sensitive to short-term change in this context, or both. These findings suggest caution in interpreting the mechanistic pathway and highlight the need for future studies to further examine both intervention dose and the suitability of psychological flexibility measures for NICU caregivers.

Contrary to our hypothesis, scores on the PSS:NICU increased in all groups from baseline to 2 weeks, as did perceived stress scores, and there was no significant difference between groups. This rise in stress underscores the need to better understand stress trajectories in parents of extremely and very preterm infants. Parents in qualitative research have described this experience as a “rollercoaster” with multiple peaks of stress [33], which may blunt intervention effects. This may be of particular relevance for digital interventions, which tend to have small to moderate effect sizes in postpartum intervention studies [12].

While NICU-specific stress was a clinically meaningful primary outcome, it may not have been the most conceptually proximal outcome for an ACT-based intervention. ACT is designed to influence psychological flexibility and responses to distress, rather than directly reduce external stressors such as those inherent to the NICU environment. Future studies may therefore benefit from considering broader distress, well-being, or coping-related outcomes as primary endpoints.

Another consideration is that although the 3-arm design allowed examination of the potential added value of ACT beyond educational content, it also increased complexity and reduced power for pairwise comparisons. Furthermore, the active control comparison arm was not a true absence of intervention: participants completed a structured 7-module digital course drawing on existing hospital web content. This active control design may have attenuated between-group differences and is an important consideration when interpreting the null findings.

Despite the absence of between-group differences on primary and secondary psychological outcomes, both digital interventions demonstrated high feasibility and acceptability. Engagement with the interventions was strong, with most intervention participants completing the interventions and rating them as “very helpful.” These findings indicate that brief digital supports can be successfully delivered to caregivers during NICU admission, even in the context of substantial emotional and practical demands.

Both digital intervention groups had higher odds of full breastfeeding at discharge compared with active control. These findings should be interpreted cautiously, as the study was not powered to detect smaller effects or differences in secondary outcomes. However, this finding aligns with evidence that shows that educational programs focused on skin-to-skin care and breastfeeding can improve breastfeeding outcomes in mothers of preterm infants [34]. Mindfulness- and relaxation-based interventions have also been found to improve breastfeeding outcomes, potentially through reductions in anxiety [35]. Both components (education and mindfulness) were included in the NPACT intervention.

Mothers in the NICU often face significant challenges in establishing breastfeeding, in part due to the prematurity of their infants [36]. Establishing breastfeeding in the NICU is important, as breast milk has been associated with lower risks of common conditions preterm infants can develop, including chronic lung disease, sepsis, and necrotizing enterocolitis [34]. These hypothesis-generating findings warrant further investigation, as brief digital educational and/or psychosocial interventions may offer a scalable, low-cost way to bolster breastfeeding rates in this vulnerable group.

Infants in the ACT-based intervention group also had lower odds of chronic lung disease compared with active control. This finding should also be interpreted with caution. The NPACT group included a higher proportion of female infants, which is associated with a lower risk of chronic lung disease compared to males [37], suggesting that baseline sex differences may be a confounding factor.

### Implications for Digital Mental Health Interventions in the NICU

In this study of caregivers of very preterm infants in the NICU, a brief digital mental health intervention was delivered at the start of acute hospitalization in a 2-week time period. The absence of between-group differences suggests that this unguided digital intervention did not significantly affect distress. This may be in part because of the acute and ongoing nature of high stress in the NICU environment, requiring an intervention with a stronger effect. Furthermore, from the perspective of ongoing and cumulative emotional, medical, and practical demands in the NICU, the intervention may be more appropriately conceptualized as seeking to attenuate or buffer an expected rise in stress, rather than produce an outright reduction in stress over the short term. This framing may better reflect the observed trajectories in the present study and help guide future trial designs.

An additional consideration when interpreting the findings is the imbalance in caregiver role across study groups, with proportionally more fathers in the active control group, alongside a higher dropout rate among fathers compared with mothers. Mothers have been shown to experience higher levels of distress than fathers in the NICU [1], and the higher proportion of fathers in the active control group may have contributed to attenuating between-group differences. No formal sensitivity analyses by caregiver role were conducted, as the study was not powered for such comparisons.

Few digital interventions of this sort have been tested with caregivers in the NICU, limiting comparisons. Generally, digital mental health interventions in perinatal populations have been found to have small to moderate effect sizes [12], and guided digital interventions (ie, delivered in combination with an in-person clinician) have been found to be more effective than unguided interventions [38] such as this one. However, the present intervention was intentionally designed to be unguided and self-directed to maximize scalability within this resource-constrained clinical setting. Importantly, these findings do not suggest that digital mental health interventions lack value for caregivers in neonatal settings; rather, they underscore the importance of aligning intervention intensity, timing, and delivery with the context and needs of the population.

Therefore, this trial highlights both the promise and limitations of an unguided digital mental health intervention in an acute clinical setting. The high levels of engagement and perceived helpfulness ratings demonstrate that caregivers are willing to use brief digital mental health interventions during NICU admission, even amidst significant stress. However, the absence of measurable psychological benefit between groups suggests that future digital interventions in similar contexts may require greater intensity, guidance, or integration within stepped-care models. Approaches such as hybrid digital–clinician support, adaptive delivery based on stress trajectories, or continuation of digital interventions beyond the acute admission phase may be more effective in addressing caregiver distress while preserving scalability. While a fully unguided format may be appropriate from an implementation and scalability perspective, future optimization work may benefit from examining whether limited human support enhances engagement and therapeutic benefit in this population.

### Strengths and Limitations

Several strengths and limitations should be considered when interpreting these findings.

At the time of study design, there were no comparable digital mental health trials conducted with caregivers in the NICU to inform effect size estimates. The study was therefore powered to detect large effects on NICU-specific parental stress using the PSS:NICU based on effect sizes reported in a prior randomized controlled trial of an in-person NICU intervention. As a result, this study may not have been sufficiently powered to detect smaller effects, including behavioral and neonatal outcomes.

Additionally, the effect size used to power this study (a reduction of 0.2 points on the PSS:NICU) was derived from a prior RCT and used as a statistical benchmark to show between-group differences, rather than an estimate of clinically meaningful change. To our knowledge, no minimal clinically important difference has been established for the PSS:NICU, which limits interpretation of the clinical significance of the null findings and represents a gap in the literature.

The interventions were unguided and relatively brief, in order to be resource-efficient and scalable; however, this may have limited their capacity to influence psychological processes or distress during periods of acute stress. Additionally, baseline imbalances, including infant characteristics, may have influenced secondary neonatal outcomes. This cohort was ethnically diverse, and further research is needed to explore how digital ACT-based and other psychosocial interventions should be culturally adapted and evaluated for effectiveness among diverse caregiver populations. Finally, findings from this single-site trial may not generalize to NICUs with different staffing models, psychosocial resources, or caregiver demographics. Nonetheless, the null findings on primary psychological outcomes are informative for the design and timing of future digital interventions in high-acuity neonatal settings.

### Conclusions

In this randomized controlled cluster trial, an unguided digital mental health intervention delivered during NICU admission did not reduce NICU-related parental stress or other psychological outcomes relative to active control. However, the digital intervention was feasible, acceptable, and highly used by participants, demonstrating that a brief digital mental health intervention can be implemented successfully in a high-acuity neonatal setting. Exploratory findings suggest potential benefits on breastfeeding, generating hypotheses for future research. These results highlight the importance of timing, intensity, and integration when designing digital mental health interventions for use in acute clinical contexts.

## Supplementary material

10.2196/92021 Multimedia Appendix 1 Additional results including per protocol analysis, change over time within groups analysis, and qualitative analysis.

10.2196/92021 Checklist 1 CONSORT checklist.

## Abbreviations

AAQ-9: Acceptance and Action Questionnaire-9
ACT: acceptance and commitment therapy
CESD-10: Center for Epidemiologic Studies Depression Scale
CONSORT: Consolidated Standards of Reporting Trials
ICC: intracluster correlation
ITT: intention-to-treat
NICU: neonatal intensive care unit
NPACT: NICU parent acceptance and commitment therapy
PSS: Perceived Stress Scale
PSS:NICU: Parental Stressor Scale: neonatal intensive care unit
STAI-6: Spielberger State Trait Anxiety Index
STAI-S: State Trait Anxiety Index-State
