# Supplementary material for: A Digital Acceptance and Commitment Therapy and Education Intervention for Caregivers of Very Preterm Infants in the Neonatal Intensive Care Unit: Randomized Controlled Trial
Source: JMIR Ment Health. 2026 Jul 23;13:e92021. doi: 10.2196/92021 (PMC13395256; doi:10.2196/92021)
Supplement: Multimedia Appendix 1 [file mental-v13-e92021-s001.docx]

Multimedia Appendix 1

Table S1: Per Protocol Linear Mixed-Model Analysis of Primary and Secondary Outcomes *(Between Groups)*

|  | Scores, Mean (SD), points | | | | | | Difference in Change Between Groups | | |
| --- | --- | --- | --- | --- | --- | --- | --- | --- | --- |
| Outcome, timepoint | Active control | n | NPACT | n | Education-only | n | *NPACT vs Active control,* *M* adjusted difference (95% CI), *P* | *Education-only vs Active control, M* adjusted difference (95% CI), *P* | *NPACT vs Education-only*, *M* adjusted difference (95% CI), *P* |
| *Primary outcome* |  |  |  |  |  |  |  |  |  |
| NICU Stress (PSS:NICU) |  |  |  |  |  |  |  |  |  |
| Baseline | 2.3 (0.9) | 33 | 2.8 (0.9) | 27 | 2.3 (1.0) | 16 | NA | NA | NA |
| 2 weeks | 2.6 (0.9) | 31 | 3.0 (0.9) | 27 | 2.6 (1.0) | 16 | 0.08 (-0.37, 0.53) *P=.*74 | -0.04 (-0.55, 0.48) *P=.*89 | 0.11 (-0.43, 0.65) *P=.*68 |
| *Secondary Outcomes* |  |  |  |  |  |  |  |  |  |
| ^a^PSS:NICU  Subscale 1 |  |  |  |  |  |  |  |  |  |
| Baseline | 1.8 (0.8) | 33 | 2.2 (0.8) | 27 | 2.0 (0.9) | 16 | NA | NA | NA |
| 2 weeks | 2.2 (0.8) | 31 | 2.5 (1.1) | 27 | 2.4 (0.9) | 16 | 0.08 (-0.43, 0.61) *P=.*75 | -0.04 (-0.54, 0.47) *P=.*89 | 0.9 (-0.42, 0.59) *P=.*74 |
| ^b^PSS:NICU  Subscale 2 |  |  |  |  |  |  |  |  |  |
| Baseline | 2.4 (1.0) | 33 | 2.9 (1.1) | 27 | 2.4 (1.0) | 16 | NA | NA | NA |
| 2 weeks | 2.6 (1.0) | 31 | 3.0 (1.2) | 27 | 2.6 (1.0) | 16 | 0.21 (-0.40, 0.82) *P=.*49 | 0.09 (-0.49, 0.69) *P=.*74 | 0.21 (-0.38, 0.80) *P=.*48 |
| ^c^PSS:NICU  Subscale 3 |  |  |  |  |  |  |  |  |  |
| Baseline | 2.7 (1.1) | 33 | 3.0 (1.0) | 27 | 2.7 (1.1) | 16 | NA | NA | NA |
| 2 weeks | 2.9 (1.1) | 31 | 3.0 (1.0) | 27 | 3.0 (1.2) | 16 | -0.9 (-0.68, 0.49) *P=.*75 | 0.02 (-0.58, 0.61) *P=.*95 | 0.04 (-0.59, 0.67) *P=.*90 |
| Perceived Stress (PSS) |  |  |  |  |  |  |  |  |  |
| Baseline | 18.0 (6.6) | 33 | 20.8 (6.9) | 27 | 19.0 (7.4) | 16 | NA | NA | NA |
| 2 weeks | 19.7 (6.2) | 31 | 21.7 (6.2) | 27 | 19.1 (6.7) | 16 | 0.99 (-2.26, 4.24) *P=.*55 | -0.84 (-4.68, 2.99) *P=.*67 | 1.83 (-2.07, 5.74) *P=.*36 |
| Discharge | 17.0 (8.1) | 25 | 17.9 (6.4) | 24 | 19.6 (6.2) | 16 | 0.37 (-3.10, 3.84) *P=.*84 | 2.70 (-1.25, 6.66) *P=.*18 | -2.33 (-6.31, 1.64) *P=.*25 |
| 3-months | 16.4 (7.4) | 24 | 16.7 (7.5) | 23 | 15.8 (5.9) | 13 | -0.05 (-3.68,3.58) *P=.*98 | -0.55 (-4.61, 3.52) *P=.*79 | 0.50 (-3.63, 4.62) *P=.*81 |
| Anxiety (STAI-6) |  |  |  |  |  |  |  |  |  |
| Baseline | 44.2 (17.2) | 33 | 50.1 (13.1) | 27 | 42.8 (15.7) | 16 | NA | NA | NA |
| 2 weeks | 45.3 (15.0) | 31 | 47.5 (14.3) | 27 | 42.2 (17.9) | 16 | -0.94 (-8.25, 6.37) *P=.*80 | -2.07 (-10.70, 6.55) *P=.*64 | 1.13 (-7.7, 9.96) *P=.*80 |
| Discharge | 41.6 (15.8) | 25 | 39.0 (13.7) | 24 | 42.1 (14.0) | 16 | -4.25 (-12.00, 3.51) *P=.*28 | 2.71 (-6.18, 11.61) *P=.*13 | -6.86 (-15.93, 2.01) *P=.*13 |
| 3-months | 39.7 (16.7) | 24 | 40.0 (13.9) | 23 | 34.9 (10.2) | 13 | -1.74 (-9.86, 6.39) *P=.*68 | -3.35 (-12.47, 5.77) *P=.*47 | 1.62 (-7.69, 10.92) *P=.*73 |
| Depression  (CESD-10) |  |  |  |  |  |  |  |  |  |
| Baseline | 10.0 (5.8) | 33 | 12.6 (6.1) | 27 | 11.1 (6.6) | 16 | NA | NA | NA |
| 2 weeks | 10.0 (6.1) | 31 | 10.2 (6.0) | 27 | 9.8 (6.5) | 16 | -1.04 (-3.63, 1.55) *P=.*43 | -0.68 (-3.69, 2.32) *P=.*66 | -0.35 (-3.43, 2.73) *P=.*82 |
| Discharge | 9.5 (6.5) | 25 | 8.9 (5.0) | 24 | 10.6 (5.9) | 16 | -1.63 (-4.40, 1.15) *P=.*25 | 0.76 (-2.35, 3.87) *P=.*63 | -2.38 (-5.53, 0.76) *P=.*14 |
| 3-months | 8.9 (6.0) | 24 | 8.5 (6.4) | 23 | 7.7 (4.5) | 13 | -1.57 (-4.48,1.34) *P=.*29 | -1.73 (-4.93,1.47) *P=.*29 | 0.16 (-3.11,3.42) *P=.*92 |
| Psychological Flexibility  (AAQ-9) |  |  |  |  |  |  |  |  |  |
| Baseline | 31.8 (7.5) | 33 | 34.1 (8.6) | 27 | 33.2 (7.4) | 16 | NA | NA | NA |
| 2 weeks | 32.9 (5.3) | 29 | 34.8 (7.2) | 26 | 31.8 (7.7) | 16 | 0.94 (-2.58, 4.46) *P=.*60 | -1.56 (-5.62, 2.50) *P=.*45 | 2.34 (-1.69, 6.36) *P=.*26 |
| Discharge | 34.6 (6.8) | 25 | 31.6 (8.5) | 23 | 33.4 (5.1) | 13 | -2.62 (-6.39, 1.15) *P=.*17 | 0.51 (-3.91, 4.94) *P=.*82 | -3.19 (-7.54, 1.16) *P=.*15 |
| 3-months | 34.7 (9.3) | 21 | 33.4 (9.5) | 21 | 31.7 (6.7) | 13 | -1.7 (-5.65, 2.26) *P=.*40 | -2.96 (-7.4, 1.47) *P=.*19 | 1.27 (-3.17, 5.70) *P=.*58 |

*Note.* NPACT = NICU Parent Acceptance and Commitment Therapy + education intervention. Outcomes analysed using linear mixed regression models with adjustment for scores at baseline, stratification factor (twin pregnancy) and a random effect for within-cluster (family) correlation. ^a^PSS:NICU Subscale 1 = Sights and Sounds of the NICU
^b^PSS:NICU Subscale 2 = Baby Behaviour and Appearance
^c^PSS:NICU Subscale 3 = Parental Role Alteration

Table S2: Summary Statistics Linear and Linear Mixed-Model Analysis of Change Over Time *(Within Groups)*

|  | Scores, Mean (SD), points | | | | | | Change Over Time Within Group | | |
| --- | --- | --- | --- | --- | --- | --- | --- | --- | --- |
| Outcome, timepoint | Active control | n | NPACT | n | Education-only | n | *Active control* *M* adjusted difference from baseline (95% CI), p value | *NPACT  M* adjusted difference from baseline (95% CI), p value | *Education-only  M* adjusted difference from baseline (95% CI), p value |
| *Primary outcome* |  |  |  |  |  |  |  |  |  |
| NICU Stress (PSS:NICU) |  |  |  |  |  |  |  |  |  |
| Baseline | 2.3 (0.8) | 36 | 2.8 (0.8) | 34 | 2.3 (0.9) | 31 | NA | NA | NA |
| 2 weeks | 2.6 (0.9) | 33 | 3.0 (0.9) | 33 | 2.5 (1.0) | 31 | 0.50 (0.16, 0.85), *P=.*005 | 0.56 (0.19, 0.94), *P=.*005 | 0.94 (0.60, 1.27), *p*<0.001 |
| *Secondary Outcomes* |  |  |  |  |  |  |  |  |  |
| ^a^PSS:NICU  Subscale 1 |  |  |  |  |  |  |  |  |  |
| Baseline | 1.8 (0.7) | 36 | 2.3 (0.7) | 34 | 1.8 (0.9) | 31 | NA | NA | NA |
| 2 weeks | 2.2 (1.4) | 33 | 2.5 (1.7) | 33 | 2.2 (1.7) | 31 | 0.57 (0.21, 0.93), *P=.*003 | 0.65 (0.20, 1.10), *P=.*006 | 0.81 (0.48, 1.14), *p*<0.001 |
| ^b^PSS:NICU  Subscale 2 |  |  |  |  |  |  |  |  |  |
| Baseline | 2.4 (1.0) | 36 | 2.9 (1.0) | 34 | 2.3 (1.3) | 31 | NA | NA | NA |
| 2 weeks | 2.7 (1.0) | 33 | 3.1 (1.1) | 33 | 2.4 (1.0) | 31 | 0.44 (0.09, 0.78), *P=.*014 | 0.65 (0.30, 0.99), *P=.*001 | 0.85 (0.54, 1.17), *p*<0.001 |
| ^c^PSS:NICU  Subscale 3 |  |  |  |  |  |  |  |  |  |
| Baseline | 2.7 (1.0) | 36 | 3.1 (1.0) | 34 | 2.8 (1.1) | 31 | NA | NA | NA |
| 2 weeks | 2.9 (1.9) | 33 | 3.1 (1.7) | 33 | 2.8 (1.4) | 31 | 0.59 (0.31, 0.88), *p*<0.001 | 0.46 (0.11, 0.80), *P=.*011 | 0.77 (0.37, 1.16), *p*<0.001 |
| Perceived Stress (PSS) |  |  |  |  |  |  |  |  |  |
| Baseline | 18.3 (6.5) | 35 | 20.4 (6.6) | 34 | 18.3 (6.3) | 31 | NA | NA | NA |
| 2 weeks | 19.8 (6.0) | 33 | 21.3 (6.0) | 33 | 18.6 (5.8) | 31 | 1.1 (-1.1, 3.3) *P=.*33 | 0.98 (-1.20, 3.15) *P=.*38 | 0.59 (-1.67, 2.85) *P=.*61 |
| Discharge | 17.0 (7.8) | 27 | 17.4 (6.7) | 30 | 17.9 (6.6) | 28 | -1.8 (-4.10, 0.49) *P=.*12 | -2.80 (-5.01, -0.55) *P=.*015 | -0.13 (-6.48, 2.22) *P=.*92 |
| 3-months | 16.5 (7.1) | 25 | 15.7 (7.4) | 26 | 15.8 (6.3) | 24 | -2.66 (-5.00, -0.13) *P=.*039 | 0.02 (-6.89, -2.12) *P=.*000 | -2.43 (-4.92, 0.07) *P=.*06 |
| Anxiety  (STAI-6) |  |  |  |  |  |  |  |  |  |
| Baseline | 44.8 (16.9) | 35 | 49.7 (13.0) | 34 | 43.5 (14.1) | 31 | NA | NA | NA |
| 2 weeks | 45.5 (14.6) | 33 | 46.8 (13.8) | 33 | 41.0 (16.1) | 31 | -0.015 (-5.03, 4.99) *P=.*99 | -3.61 (-8.57, 1.34) *P=.*15 | -1.99 (-7.12, 3.13) *P=.*45 |
| Discharge | 41.3 (15.2) | 27 | 37.9 (13.5) | 30 | 40.5 (14.3) | 28 | -4.19 (-9.37, 0.99) *P=.*11 | -12.25 (-17.38, -7.11) *P=.*000 | -2.58 (-7.92, 2.76) *P=.*34 |
| 3-months | 39.7 (16.3) | 25 | 38.2 (14.4) | 26 | 38.6 (14.2) | 24 | -6.75 (-12.33, -1.16) *P=.*018 | -13.03 (-18.62, -7.44) *P=.*000 | -5.25 (-10.92, 0.41) *P=.*07 |
| Depression  (CESD-10) |  |  |  |  |  |  |  |  |  |
| Baseline | 9.6 (5.8) | 35 | 11.9 (6.0) | 34 | 10.1 (6.1) | 31 | NA | NA | NA |
| 2 weeks | 10.0 (6.0) | 33 | 10.3 (6.0) | 33 | 8.6 (5.9) | 31 | 0.23 (-1.50, 1.95) *P=.*80 | -1.52 (-3.24, 0.20) *P=.*08 | -1.45 (-3.22, 0.33) *P=.*11 |
| Discharge | 9.5 (6.3) | 27 | 8.8 (4.9) | 30 | 9.1 (6.1) | 28 | -0.45 (-2.24, 1.34) *P=.*62 | -2.95 (-4.73, -1.16) *P=.*001 | -1.24 (-3.10, 0.61) *P=.*19 |
| 3-months | 8.9 (5.8) | 25 | 7.7 (6.1) | 26 | 7.6 (5.5) | 24 | -0.57 (-2.53, 1.38) *P=.*57 | -1.29 (-6.39, -2.49) *P=.*000 | -2.66 (-4.63, -0.69) *P=.*008 |
| Psychological Flexibility  (AAQ-9) |  |  |  |  |  |  |  |  |  |
| Baseline | 32.0 (7.3) | 35 | 34.1 (8.4) | 34 | 32.3 (6.9) | 31 | NA | NA | NA |
| 2 weeks | 33.5 (5.6) | 31 | 34.7 (6.8) | 32 | 30.8 (7.0) | 29 | 0.89 (-1.55, 3.33) *P=.*47 | 0.15 (-2.24, 2.55) *P=.*90 | -0.71 (-3.25, 1.85) *P=.*59 |
| Discharge | 34.8 (6.6) | 27 | 32.2 (8.1) | 28 | 31.3 (7.2) | 23 | 1.02 (-1.49, 3.52) *P=.*43 | -2.18 (-4.70, 0.35) *P=.*09 | 1.42 (-1.39, 4.24) *P=.*32 |
| 3-months | 34.7 (8.9) | 23 | 33.2 (9.1) | 25 | 32.5 (10.4) | 22 | 1.88 (-0.92, 4.67) *P=.*19 | -1.70 (-4.43, 1.03) *P=.*22 | 1.64 (-1.23, 4.50) *P=.*26 |

*Note.* NPACT = NICU Parent Acceptance and Commitment Therapy + education intervention. Outcomes analysed using linear mixed regression models with adjustment for scores at baseline, stratification factor (twin pregnancy) and a random effect for within-cluster (family) correlation. ^a^PSS:NICU Subscale 1 = Sights and Sounds of the NICU
^b^PSS:NICU Subscale 2 = Baby Behaviour and Appearance
^c^PSS:NICU Subscale 3 = Parental Role Alteration

# Table S3: Qualitative Feedback on the NPACT Digital Intervention

| Sentiment | Theme | Prevalence  (n = 28) | Illustrative Quote |
| --- | --- | --- | --- |
| Positive | Overall helpfulness | 15 (54%) | “Extremely helpful while going through the stress of being in NICU.” (F6) |
|  | ACT-based strategies (general) | 15 (54%) | “I appreciate the mindfulness and breathing exercises. I will remind myself to use them when I need.” (M5) |
|  | Breathing and grounding exercises | 10 (36%) | “The breathing exercises were most helpful… I think it helped.” (M14) |
|  | Connecting with baby | 4 (14%) | “Knowing the different ways I can connect with my little one.” (F1) |
|  | Education content (general) | 17 (61%) | “The overall course was extremely helpful… it helped me understand a lot more about cares and spending time with your baby.” (M6) |
|  | Skin-to-skin care | 14 (50%) | “Even though I'm not breastfeeding, I learned why it's important to do skin-to-skin.” (M18) |
|  | Expressing and breastfeeding information | 13 (46%) | “Everything is helpful: steps on how to pump and store milk; partner’s support.” (M10) |
|  | Understanding infant cues and development | 10 (36%) | “So good to understand baby’s different moods and signs.” (M17) |
| Negative/ Challenges | Mindfulness activities did not resonate | 6 (21%) | “Watching the mindfulness videos… they were not something that interests me.” (W1) |
|  | Difficulty focusing or engaging | 4 (14%) | “I could not keep focus.” (M16) |
|  | Emotional difficulties | 3 (11%) | “I found the mindfulness activity tricky… I feel so much guilt at the moment.” (M6) |
|  | Information overload | 3 (11%) | “Too much info.” (M18) |
|  | Skin-to-skin not yet possible | 2 (7%) | “Skin-to-skin is not happening for us yet, but we are looking forward to it.” (F5) |

Note. Qualitative responses were provided by 28 participants (M = mother, F = father, W = whānau/extended family member). Percentages reflect the proportion of respondents endorsing each theme; participants could endorse multiple themes. Only themes that were quantified in the original qualitative coding framework are presented.
